# Response of FDG avid pelvic bone marrow to concurrent chemoradiation for anal cancer

**DOI:** 10.1016/j.radonc.2019.08.016

**Published:** 2020-02

**Authors:** Maxwell Robinson, Rebecca Muirhead, Clare Jacobs, Rosie Cooke, Kwun-Ye Chu, Frank Van den Heuvel, Stasya Ng, Pradeep Virdee, Victoria Strauss, Maria Hawkins

**Affiliations:** a CRUK/MRC Oxford Institute for Radiation Oncology, University of Oxford, UK; b Department of Oncology, Oxford University Hospitals NHS Trust, UK; c Oncology Clinical Trials Office, University of Oxford, UK; d Centre for Statistics in Medicine, University of Oxford, UK

**Keywords:** Haematological toxicity, Pelvic bone marrow, Intensity modulated radiotherapy, Positron-emission tomography

## Abstract

**Background and purpose:**

To determine if suppression of active bone marrow, as defined on FDG PETCT, is seen in on-treatment imaging of anal cancer patients receiving concurrent chemoradiation.

**Methods and materials:**

Scans from 26 patients participating in the ART trial (full title: Anal squamous cell carcinoma: Investigation of functional imaging during chemoRadioTherapy), a single center observational study with FDG PETCT prior to radiotherapy and at fraction 8–10 of concurrent chemoradiation were analysed. Active bone marrow was contoured in both the pelvis and un-irradiated thoracic spine. SUV and volume of active bone marrow after 8–10 fractions of treatment were compared to baseline. Dose metrics to pelvic active bone marrow were extracted and compared to reduction in SUV/active bone marrow volume and to blood count nadir using linear regression.

**Results:**

Suppression of active bone marrow is seen in the pelvis by a reduction in mean SUV and volume of active bone marrow after 8–10 fractions of treatment. Suppression is not seen in un-irradiated thoracic spine. Dose metrics were associated with reduced SUV and reduced volume of active bone marrow. Volume of active bone marrow receiving <20 Gy was associated with WCC/ANC nadir. 20 Gy was identified as the most likely clinically meaningful dose threshold for toxicity. Volume of active bone marrow receiving <20 Gy correlated to WCC and ANC with an increase of 100 cc being associated with an increase of 0.4 and 0.3 respectively.

**Conclusion:**

The effect of concurrent chemoradiation in suppression of active bone marrow is seen in on-treatment FDG PETCT scans. Chemotherapy appears well tolerated after 2 weeks of treatment.

## Highlights

• Chemoradiation suppression of active bone marrow shown in on-treatment FDG-PET.
• No suppression shown in un-irradiation bone marrow.
• Volumes of active bone marrow receiving 20 Gy are associated with blood count nadirs.

Radical chemoradiation is the standard treatment in loco-regional anal cancer, achieving a 3 year disease free survival of 73% [1], [2], [3]. Intensity modulated radiotherapy (IMRT) is now used as standard in the UK [1]. IMRT enables the delivery of varying dose levels to multiple targets while decreasing dose to organs at risk resulting in reduced adverse events [4]. Additionally, IMRT offers the ability to spare pelvic bone marrow (PBM) but this is not routinely done for anal cancer. Irradiation of PBM is associated with increased risk of haematological toxicity (HT), the dose threshold for PBM survival is unclear but a number of studies have shown association with toxicity at the level of 10–20 Gy [5], [6]. A recent UK audit reported HT grade 3 or greater of 18% using IMRT [7]. Whilst sparing of bone marrow as a whole at the crucial 10–20 Gy dose level is unachievable due to the overlap of PTVs a degree of sparing of bone marrow is achievable [8], [9]. Studies have shown blood count nadir to correlate more strongly with particular sub structures (iliac and lumbosacral) of PBM [5], [10], which is evidence of the potential effectiveness of targeted sparing. In addition, it is known that PBM itself is not homogeneous and is divided into hematopoietically active and inactive regions. If sparing can be directed to active PBM this may have a significant impact on blood count nadirs and subsequent rates of acute haematological toxicity. Functional imaging studies have been published identifying active PBM [11], [12]. Evidence suggests ^18^F-Fluorothymidine (FLT) PETCT best identifies active PBM regions but is not a routinely performed scan in the UK. ^18^F-Fludeoxyglucose (FDG) PETCT is, however, widely available. Active PBM volumes identified on FDG PETCT have been shown to be more variable than FLT PETCT defined volumes but to also correlate strongly [13]. It has been shown irradiation to regions of high uptake on FDG to associate with HT whereas regions of low uptake did not in cervical cancer patients [14]. However, a recent study in anal cancer patient failed to show any improvement in association of active regions of bone marrow relative to whole bone in anal cancer patients [15]. As such, the link between FDG uptake and hematopoietic activity/HT is unclear.

We hypothesize that the effects of chemotherapy and chemoradiation (CRT) in suppressing active bone marrow pre and during CRT for anal cancer can be quantified and patients that are likely to develop HT can potentially be identified with a PETCT in the second week of CRT

Here we report an exploratory endpoint studied in a prospective observational study

## Methods and materials

### Patient selection and imaging

26 patients with anal cancer that have been prospectively enrolled into the ART trial (full title: Anal squamous cell carcinoma: Investigation of functional imaging during chemoRadioTherapy (approved by local institution ethics board)) have been investigated. In brief, the ART trial is a prospective observational single centre study evaluating the role of functional imaging during radical CRT for anal cancer. As part of the study procedure patients undergo PETCT at 2 time points (baseline and at fraction 8–10 of treatment (wk2 scan)). 20 patients had paired PETCT scans. 12 patients had wk2 scans of whole body; the remaining 8 patients had scan range limited to primary tumour. Eligibility criterial included confirmed invasive primary squamous carcinoma of the anus, stage T2N0 or greater, did not have prosthetic hip and were radiotherapy naive. All patients had PETCT scans on either a GE Discovery 690 (*n* = 4) or 710 PETCT (*n* = 22) scanners. Patients were injected with 4 MBq per kg of body weight up to a maximum of 600 MBq and scanned a minimum of 60 minutes post injection (75 min on average). Time from injection to scan (FDG uptake time) was recorded. Scans were performed at 3.75 mm slice thickness with 4 min PET acquisition per bed position. Rigid registration was performed to radiotherapy planning CT using bony match in Eclipse v11 (Varian Medical Systems) image registration module. PETCT scanners was subjected to regular QA including SUV calibration.

### Treatment

#### Radiotherapy

Patients were treated using 7–9 field IMRT or coplanar RapidArc in 28 fractions using simultaneous integrated boost. Delineation was as per UK guidance [1]. In summary, gross anal tumour plus a 2.5 cm margin received either 53.2–61.6 Gy (if T3 and T4) or 41.4–50.4 Gy in 23-28F (if T2); the involved nodes plus a 2 cm margin received 50.4 Gy and the prophylactic nodes (plus 0.5–1 cm margin) received 34.5–40 Gy in 23-28F. A constraint was placed on femoral head dose (dose to 50% less than 30 Gy, dose to 35% less than 40 Gy and dose to 5% less than 44 Gy) but dose to other pelvic bone structures was unconstrained.

#### Chemotherapy

Patients fit enough for concurrent chemotherapy were planned to receive 12 mg/m2 Mitomycin D1 and 825 mg/m2 bd Capecitabine D1-D28 radiotherapy days only. Capecitabine was withheld with thrombocytopenia Grade 2 or neutropenia Grade 3 or any Grade 3 non-haematological toxicity considered related to Capecitabine; until it resolved to G1 then restarted at the same dose or at a reduced dose.

### Bone marrow delineation

Pelvic bone marrow (PBM) was delineated using the external surface of bone and sub divided into three sub structures; iliac BM, extending from the iliac crest to the superior edge of femoral head, lower pelvis BM, extending from the superior edge of femoral heads and including all pelvic bone as well as proximal femoral bone down to the level of and including the inferior ischial tuberosities, and lumbosacral BM, extending from the level of the superior border of L5 to the superior edge of femoral heads. Sub division of BM was based on previously published work by Mell et al. [6], [10]. Un-irradiated vertebra (T9–11) were also delineated. For ease and consistency of contouring only the vertebral bodies were contoured.

### Standard uptake values

The mean SUV of PBM, PBM sub structures and un-irradiated vertebra were extracted at both baseline and wk2 scans. To avoid the influence of temporal changes in the uptake of FDG, SUV analysis was omitted for patients with a greater than 20 minutes difference in uptake time between baseline and wk2 scans.

### Active bone marrow delineation and dose metrics

Active bone marrow (activeBM) was defined using a threshold of SUV greater than the mean SUV seen in PBM as a whole for each individual patient at baseline. ActiveBM volumes were created for all PBM sub structures for baseline and wk2 scans. The CT component of the PETCT was rigidly registered to the planning CT and activeBM structures transferred to planning CT. Total treatment mean dose (Gy) and volumes receiving 10 to 45 Gy, in 5 Gy increments (V10-45), were extracted for activeBM structures.

### Bloods

White cell count (WCC) including absolute neutrophil count (ANC) were collected at baseline and weekly during chemoradiation. Analysis endpoints were blood counts nadirs.

### Analysis

For patients with evaluable wk2 scans, mean SUV and absolute volumes of activeBM were compared using paired Wilcoxon signed test to that at baseline. Dose metrics and baseline values were compared to the percentage change in SUV and percentage change in volume of activeBM using linear regression analysis. Linear regression was chosen as it allows us to quantify the effect of the dosage on outcomes and obtain preliminary effect estimates to see if there are signals to suggest associations. To ensure linear regression was suitable, regression assumptions were assessed using plots of normality and residual analysis, see Supplementary Fig. 1. For all patients, baseline volume of active bone marrow receiving less than a threshold dose for toxicity was then compared to ANC and WCC nadir (both absolute and as a ratio of baseline), again using linear regression analysis. A two-sided 5% significance level was used.

## Results

12 patients with paired PET scans were available. Patients received radiotherapy doses of 50.4 Gy (4 patients); 53.2 Gy (6 patients) and 61.6 Gy (2 patients) in 28 fractions. One patient received 41.4 Gy in 23 fractions. In a single patient receiving 50.4 Gy, FDG uptake time differed by 30 minutes. This patient was subsequently excluded from SUV analysis. In all other patients, uptake time was on average 5 minutes different between scans (range: 0–16 min).

Median SUV values are shown in Table 1. There was a statistically significant reduction in the median SUV in the PBM from baseline to wk2 scan (median difference of −0.20 (95% CI: −0.29, −0.04), *p* = 0.006). A decrease in mean SUV was observed in all substructures with the largest decrease seen in lumbosarcral (−0.22 (−0.41, −0.07) and roughly equal reduction in iliac (−0.16 (−0.31, 0.01) and lower pelvis (−0.16 (−0.27, −0.01). A reduction was not observed in the non-irradiated thoracic spine (0.05 (−0.25, 0.19)).

**Table 1:** SUV values for PBM and PBM sub structure at baseline and mid-treatment scan.

Mean SUV Values						
		PBM	Illiac BM	Lower Pelvis BM	Lumbosacral BM	T9-11
*Baseline*						
	Median	1.13	1.17	1.00	1.33	1.66
	Max	1.63	1.69	1.40	2.07	2.70
	Min	0.83	0.90	0.73	0.90	1.28

*Mid-Treatment*						
	Median	0.99	1.09	0.85	1.16	1.67
	Max	1.23	1.33	1.09	1.43	2.37
	Min	0.74	0.74	0.64	0.79	0.45

*Abbreviations:* BM = Bone Marrow; PBM = Pelvic Bone Marrow; SUV = Standard Uptake Value; T9-11 = Thorax Spine Vertebra 9-11.

**Fig. 1:**
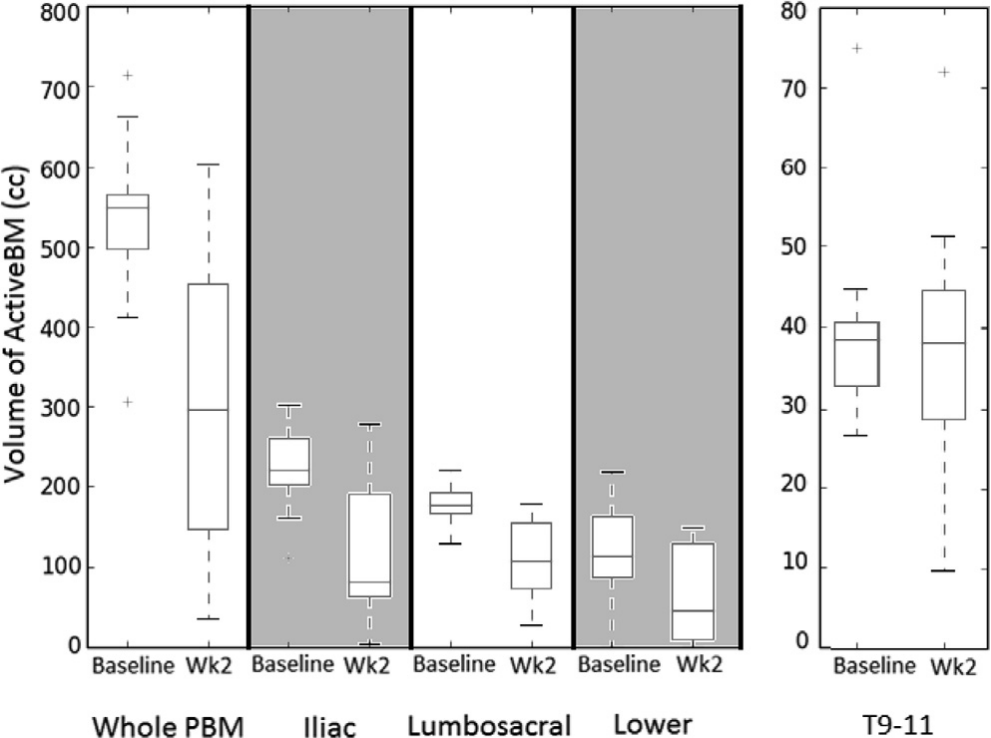
Absolute activeBM volumes at baseline and mid-treatment for PBM, PBM sub structures and un-irradiation thoracic spine.

The average volume of activeBM expressed as a percentage of whole bone structure at baseline and wk2 is shown in Table 2. Fig. 1 show boxplots of all patient data. Volumes showed a statistically significant reduction at mid-treatment in iliac and lumbosacral activeBM. Lower pelvic activeBM was strongly suggestive (*p* < 0.1) only. Again non-significant change was observer in non-irradiated thoracic spine. Baseline and wk2 activeBM for a single patient is shown in Supplementary Fig. 2.

**Table 2:** Mean volumes of activeBM.

ActiveBM Volumes					
	PBM	illiac	lumbosacral	lower	T9-11
*Baseline*					
Percentage of Whole Bone	40%	48%	57%	23%	46%
Absolute (cc)	533	225	180	128	40

*Mid-treatment*					
Percentage of Whole Bone	23%	26%	36%	12%	43%
Absolute (cc)	298	123	112	63	37

*Abbreviations:* BM = Bone Marrow; PBM = Pelvic Bone Marrow; T9-11 = Thorax Spine Vertebra 9-11.

For lower pelvis active bone marrow V10 was 100% for all case, V15 was 100% in all but 2 cases and V20 all but 4 cases, Supplementary Table 1 shows all active bone marrow dose metrics. Regression analysis was subsequently performed for iliac and lumbosacral active bone marrow at all dose levels but for lower pelvis at V25-45 only. For V20, both iliac and lumbosacral bone marrow dose metrics were associated with percentage change in SUV; (iliac V20 −0.7 (95% CI: −1.2 to −0.1), *p* = 0.026; lumbosacral V20 −0.3 (95% CI: −0.6 to −0.1), *p* = 0.043) and percentage change in volume of ActiveBM in wk2 scan (iliac V20 −2.3 (95% CI: −4.2 to −0.5), *p* = 0.017; lumbosacral V20 −0.9 (95% CI: −1.5 to −0.3), *p* = 0.011). Further results are provided in Table 3. V25 also showed statistical significance for both iliac and lumbosacral bone marrow to reduction in SUV and activeBM. Dose metrics for lower pelvis were not statistically significantly associated with a reduction in SUV but did show a statistically significant association with a reduced in activeBM volume for V25-30.

**Table 3:** Uni-variant linear regression analysis results of activeBM dose metrics and the relative reduction seen in volume of activeBM at mid-treatment.

Reduction in mean SUV			
Input variable	Coefficient	95% CI	*p*-value
Baseline iliac SUV	−16.9	−44.6 to 10.7	0.199
V20 dose	−0.7	−1.2 to −0.1	0.026
Constant	42.2	−4.2 to 88.5	0.070
Baseline lumbo SUV	−18.5	−39.9 to 2.8	0.081
V20 dose	−0.3	−0.6 to −0.1	0.043
Constant	25.1	−5.6 to 55.7	0.097

Reduction in Volume of ActiveBM			
Input variable	Coefficient	95% CI	*p*-value
Baseline iliac volume	−23.0	−114.8 to 68.7	0.584
V20 dose	−2.3	−4.2 to −0.5	0.017
Constant	107.7	−46.2 to 261.6	0.148
Baseline lumbo volume	−24.4	−69.0 to 20.1	0.246
V20 dose	−0.9	−1.5 to −0.3	0.011
Constant	40.7	−23.3 to 104.8	0.184

*Abbreviations:* BM = Bone Marrow; CI = Confidence Interval; SUV = Standard Uptake Value; V20 = Volume Receiving 20Gy.

**Fig. 2:**
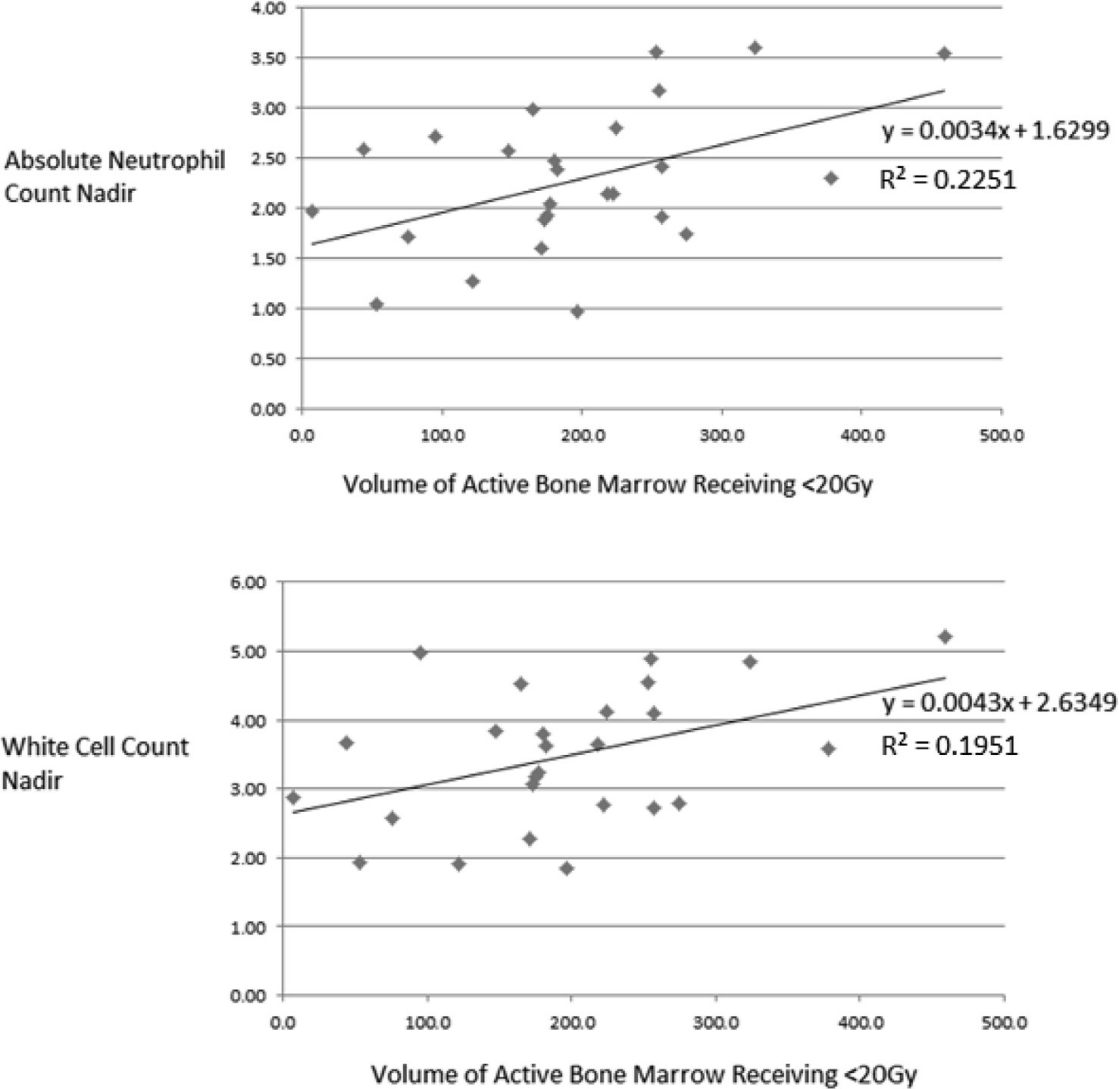
Volume of activeBM receiving less than 20 Gy versus ANC/WCC nadir.

As the lower dose level, 20 Gy was chosen as most likely threshold for clinically meaningful toxicity. Regression analysis of the volume of activeBM receiving less than 20 Gy to WCC and ANC nadir are shown in Table 4 and all data with linear fit can be seen in Fig. 2 (ANC and WCC data for all patient are shown in Supplementary Table 2). Volume receiving less than 20 Gy showed a statistically significant association to blood count nadir at a *p* value of <0.001. On initial investigation R squared values were shown to be lower when comparing dose metrics to nadir as a ratio of baseline (<0.1), i.e. corrected values. Whilst it is not clear why this is the case it suggests the relationship between baseline values and nadir is complex.

**Table 4:** Uni-variant linear regression analysis results of activeBM volume receiving less than 20 Gy and ANC/WCC nadir during chemoradiation.

ActiveBM volume receiving <20 Gy against ANC nadir			
Input variable	Coefficient	95% CI	*p*-value
V20 dose	0.0034	0.0007–0.0060	0.014
Constant	1.6299	1.0523–2.2076	<0.001

ActiveBM volume receiving <20 Gy against WCC nadir			
Input variable	Coefficient	95% CI	*p*-value
V20 dose	0.0043	0.0006–0.0080	0.024
Constant	2.6349	1.8238–3.4459	<0.001

*Abbreviations:* ANC = Absolute Neutrophil Count; BM = Bone Marrow; CI = Confidence Interval; WCC = White Cell Count; V20 = Volume Receiving 20Gy.

## Discussion

This prospective study looked to determine if the effect of chemoradiation on pelvic bone marrow could be quantified early using FDG-PETCT scans of anal cancer patients after 8–10 fractions of radiotherapy and potential implication in nadir of blood counts. To our knowledge this has not been previously reported. The effect of chemotherapy was isolated from chemoradiation by looking at an un-irradiated vertebra. The combined effect of chemoradiation is readily apparent in a reduction in SUV values of PBM (median reduction of 0.2 (16%) from baseline) with strong statistical significance (*p* < 0.01). The effect of chemoradiation is also apparent in the volumes of activeBM which were significantly reduced; iliac and lumbosacral activeBM showed a greater than 20% loss when expressed as a percentage of whole BM. The distribution of pelvic activeBM shown in this study is consistent with previous publications [16] with the majority located in iliac and lumbosacral bone. Only 23% was found in the lower pelvis, this combined with the fact lower pelvic bone receives a very high dose (V20 > 85%, the highest dose of any PBM sub structure) would indicate the significance of any sparing of lower pelvic bone on rates of haematological toxicity can be largely discounted. This is contrast to a recent study [17] where association was shown between whole lower pelvis bone marrow volume receiving 40 Gy and toxicity rates, suggesting either confounding factors not included in multivariate analysis in the study mentioned are associated with the volume of lower pelvic bone marrow receiving 40 Gy or a more complex relationship between FDG uptake and hematopoietic activity. Further investigation is warranted but is beyond the scope of this work. The effect of chemotherapy only in this study as shown in un-irradiated vertebra did not show any impact on SUV suggesting chemotherapy has less of an effect on the active bone marrow in the initial 2 weeks of treatment. As blood count nadir typically present at week 3–4 of treatment [5], [17], corresponding to fraction 11–20, suppression of active bone marrow in CRT for anal cancer is therefore likely the combined toxicity of chemoradiation.

The association of Iliac and lumbosacral activeBM dose metrics to both the relative reduction in SUV and relative reduction in activeBM volumes shows the suppression of activeBM seen in FDG PET is related to the dose received, with volumes receiving 20 Gy showing the most likely threshold for clinically meaningful toxicity. Volume of activeBM receiving less than 20 Gy showed statistically significant association to WCC and ANC nadir, with an additional 100 cc of sparing correlating to 0.3 and 0.4 diminishing ANC and WCC nadir respectively. Sparing directed at activeBM in iliac and lumbosacral bone at the 20 Gy level is achievable and may be beneficial in reducing rates of haematological toxicity.

The main limitation of this work is a relatively small sample size, subsequently whilst we have attempted to quantify the relationship between dose to activeBM and blood count nadir this should be strongly viewed in the context of the small sample size, larger studies are required. Additionally, whilst the definition of activeBM as that greater than the mean SUV seen in an individual patients whole PBM is used here and elsewhere in literature [16] this is largely a pragmatic arbitrary threshold rather than one based on physiology.

In summary, the impact of concurrent chemoradiation on PBM is seen in reduced SUV values 8–10 fractions into treatment compared to baseline. Conversely, the effect of chemotherapy only on SUV appears non-significant after 8–10 fractions into treatment. 20 Gy is shown to be the most likely threshold for toxicity and volume of activeBM receiving less than 20 Gy has been shown to correlate to ANC and WCC nadir. When sparing to protect pelvic bone marrow, clinical practice should consider sparing targeted at FDG defined activeBM volumes in the iliac and lumbosacral bone. However, it is unknown if in practice sparing of whole bone would be equally effective as sparing directed to activeBM. Larger scale studies are required.

## Declaration of Competing Interest

The authors declare that they have no known competing financial interests or personal relationships that could have appeared to influence the work reported in this paper.
