# Supplementary material for: Response of FDG avid pelvic bone marrow to concurrent chemoradiation for anal cancer
Source: Radiother Oncol. 2020 Feb;143:19–23. doi: 10.1016/j.radonc.2019.08.016 (PMC7077746; doi:10.1016/j.radonc.2019.08.016)
Supplement: Supplementary data 1 [file mmc1.docx]

**Supplementary Table One:** Active bone marrow dose metrics

| Volume (cc) | V10 | V15 | V20 | V25 | V30 | V35 | V40 | V45 |
| --- | --- | --- | --- | --- | --- | --- | --- | --- |
| Active illiac bone marrow | | |  |  |  |  |  |  |
| 258.4 | 74.8 | 67.1 | 62.5 | 55 | 42.7 | 28.8 | 18.6 |  |
| 263.5 | 54 | 49.3 | 46.3 | 41.6 | 36.4 | 27.3 | 8.5 |  |
| 193.1 | 100 | 93.5 | 81 | 68.9 | 49.8 | 31.5 | 6.3 |  |
| 264.1 | 74.2 | 62.2 | 53.7 | 42 | 34 | 24.3 | 5.9 |  |
| 225.8 | 64.4 | 54.6 | 49.6 | 43.9 | 28.9 | 8.7 | 0.5 |  |
| 205.4 | 51.8 | 45.5 | 39.8 | 35.1 | 29.8 | 19.7 | 2.7 |  |
| 213.1 | 66.4 | 59.7 | 54.1 | 47.4 | 39.3 | 24.3 | 4.4 |  |
| 301.4 | 63.6 | 58.8 | 55.6 | 47.1 | 33.3 | 14.9 | 2.8 |  |
| 232.8 | 64.9 | 59.6 | 54.8 | 45.9 | 34.2 | 15.5 | 0 |  |
| 111.7 | 64.1 | 51.7 | 45.9 | 41.2 | 32.9 | 13.9 | 1.4 |  |
| 216.5 | 56.8 | 50.6 | 42.6 | 22.9 | 7.3 | 0 | 0 |  |
| 160 | 63.8 | 55.3 | 50.5 | 41.7 | 26.7 | 8.6 | 0.5 |  |
|  |  |  |  |  |  |  |  |  |
| Active lumbosacral bone marrow | | | |  |  |  |  |  |
| 196.7 | 72.2 | 65.7 | 59.8 | 55 | 42.4 | 28.8 | 18.8 |  |
| 221.2 | 54 | 49.3 | 45.5 | 43.2 | 40.1 | 36.4 | 23 |  |
| 191.9 | 100 | 100 | 100 | 100 | 100 | 88.7 | 54.7 |  |
| 170.2 | 71.6 | 65 | 59.7 | 55.4 | 48.7 | 39 | 16.1 |  |
| 164.4 | 62.8 | 54.6 | 45.6 | 37.1 | 27.7 | 17.4 | 3.5 |  |
| 129 | 12.6 | 9.3 | 7.3 | 7.3 | 7.2 | 6.3 | 1.7 |  |
| 167.3 | 69.6 | 64.1 | 58.7 | 53 | 47.2 | 38.9 | 15 |  |
| 173.6 | 48.3 | 42.4 | 38.5 | 35.5 | 29 | 22.2 | 7.9 |  |
| 181.5 | 47.7 | 43.5 | 40.6 | 39.3 | 33.5 | 21.8 | 7.8 |  |
| 189 | 60.8 | 54.3 | 49.6 | 45.6 | 38.6 | 30 | 12.6 |  |
| 199.9 | 50.3 | 43.1 | 36.9 | 32.2 | 22.5 | 4.4 | 0 |  |
| 164 | 64.7 | 57.9 | 52.2 | 46.9 | 40 | 29.9 | 12.5 |  |
|  |  |  |  |  |  |  |  |  |
| Active lower pelvis bone marrow | | | |  |  |  |  |  |
| 113.5 | 100 | 100 | 100 | 99.7 | 96.3 | 84.1 | 60.3 | 22.5 |
| 218.2 | 100 | 100 | 100 | 96.7 | 82.6 | 67 | 17.2 | 0.8 |
| 146.2 | 100 | 100 | 100 | 99 | 90.5 | 79.3 | 42.8 | 9.6 |
| 109.7 | 100 | 100 | 100 | 97.9 | 87.8 | 68.9 | 12.4 | 0.5 |
| 157.7 | 100 | 100 | 93.5 | 86.3 | 82.1 | 69.9 | 31.2 | 9.6 |
| 208.6 | 100 | 100 | 100 | 94.1 | 85.5 | 69.5 | 35.8 | 14.4 |
| 82.1 | 100 | 100 | 100 | 97.5 | 76.3 | 40 | 7.6 | 0.5 |
| 180.1 | 100 | 100 | 97.9 | 93.6 | 81.7 | 83 | 19.3 | 8.5 |
| 114.3 | 100 | 97.4 | 94.6 | 85.4 | 65 | 33.7 | 9.4 | 1.9 |
| 0 | No bone marrow above threshold in lower pelvis | | | | |  |  |  |
| 79.6 | 100 | 98.3 | 88.7 | 55.1 | 26.2 | 5.3 | 0 | 0 |
| 88.8 | 100 | 100 | 100 | 94.8 | 83.5 | 54.6 | 20.1 | 9.3 |
