# Supplementary material for: Response of FDG avid pelvic bone marrow to concurrent chemoradiation for anal cancer
Source: Radiother Oncol. 2020 Feb;143:19–23. doi: 10.1016/j.radonc.2019.08.016 (PMC7077746; doi:10.1016/j.radonc.2019.08.016)
Supplement: Supplementary data 2 [file mmc2.docx]

**Supplementary Table Two:** Baseline and nadir ANC and WCC counts for each patient in the study, counts are 10^9^/litre.

| **Patient** | **Blood Baseline (ANC)** | **Blood Nadir** | **Blood Baseline (WCC)** | **Blood Nadir** |
| --- | --- | --- | --- | --- |
| 1 | 4.24 | 1.27 | 6.14 | 1.90 |
| 2 | 2.86 | 1.72 | 5.08 | 2.57 |
| 3 | 4.76 | 2.39 | 8.61 | 3.63 |
| 4 | 11.4 | 1.97 | 11.93 | 2.87 |
| 5 | 3.73 | 2.42 | 8.97 | 4.09 |
| 6 | 4.7 | 2.47 | 8.60 | 3.80 |
| 7 | 9.1 | 2.99 | 12.00 | 4.53 |
| 8 | 3.63 | 2.57 | 6.06 | 3.83 |
| 9 | 3.2 | 1.74 | 5.29 | 2.78 |
| 10 | 6.5 | 2.72 | 9.19 | 4.97 |
| 11 | 3.7 | 2.59 | 6.41 | 3.67 |
| 12 | 3.6 | 1.60 | 6.20 | 2.27 |
| 13 | 3.1 | 0.97 | 4.69 | 1.85 |
| 14 | 7.1 | 2.30 | 9.93 | 3.58 |
| 15 | 6.6 | 2.15 | 8.53 | 2.76 |
| 16 | 9.8 | 3.60 | 14.50 | 4.85 |
| 17 | 4.6 | 3.56 | 7.06 | 4.54 |
| 18 | 4.6 | 3.54 | 7.48 | 5.21 |
| 19 | 4.5 | 2.05 | 8.03 | 3.23 |
| 20 | 5.2 | 1.91 | 6.68 | 2.72 |
| 21 | 8.6 | 2.14 | 14.70 | 3.65 |
| 22 | 7.0 | 2.80 | 10.62 | 4.12 |
| 23 | 6.5 | 1.04 | 9.36 | 1.93 |
| 24 | 6.1 | 3.18 | 10.06 | 4.89 |
| 25 | 3.9 | 1.93 | 7.02 | 3.17 |
| 26 | 5.88 | 1.89 | 8.85 | 3.07 |
