# Supplementary material for: Response of FDG avid pelvic bone marrow to concurrent chemoradiation for anal cancer
Source: Radiother Oncol. 2020 Feb;143:19–23. doi: 10.1016/j.radonc.2019.08.016 (PMC7077746; doi:10.1016/j.radonc.2019.08.016)

(A) Plot of normality

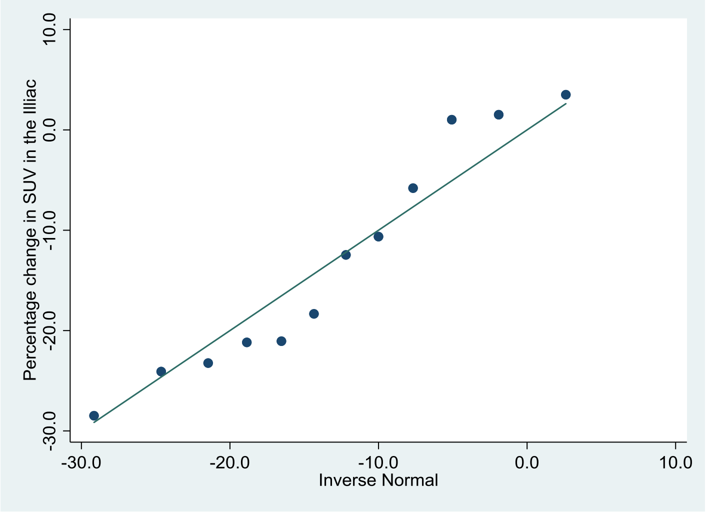

(B) Scatterplot of outcome vs predictor

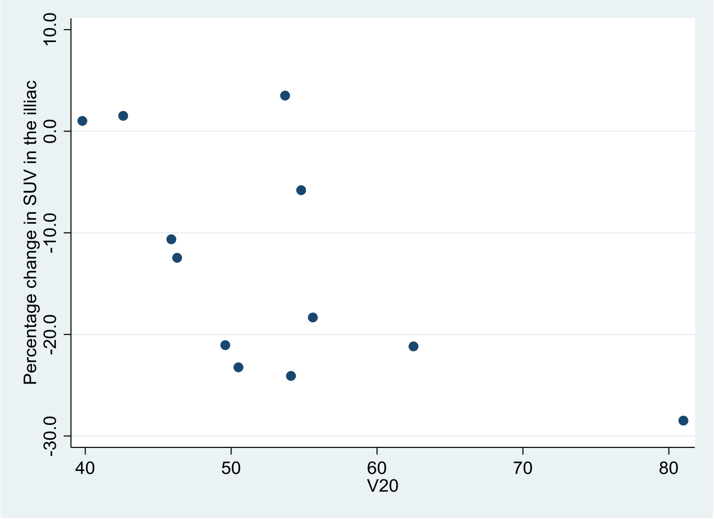

(C) Scatterplot of residuals vs fitted values

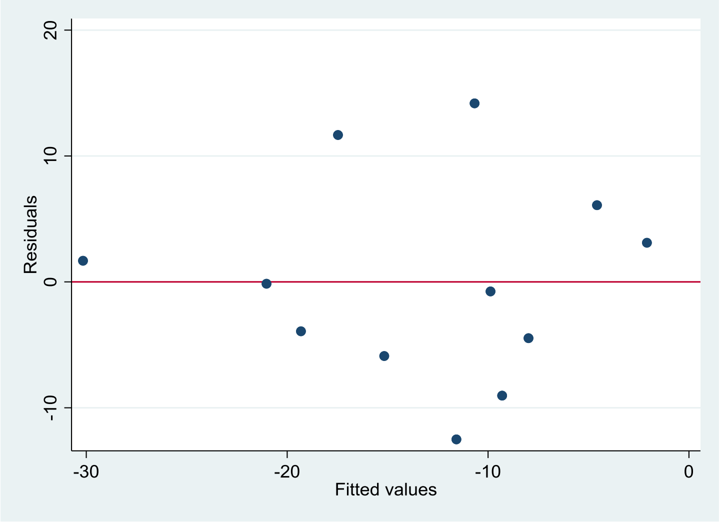

Supplement: Supplementary data 3 [file mmc3.pdf]
