# Supplementary figures and images for: Response of FDG avid pelvic bone marrow to concurrent chemoradiation for anal cancer
Source: Radiother Oncol. 2020 Feb;143:19–23. doi: 10.1016/j.radonc.2019.08.016 (PMC7077746; doi:10.1016/j.radonc.2019.08.016)

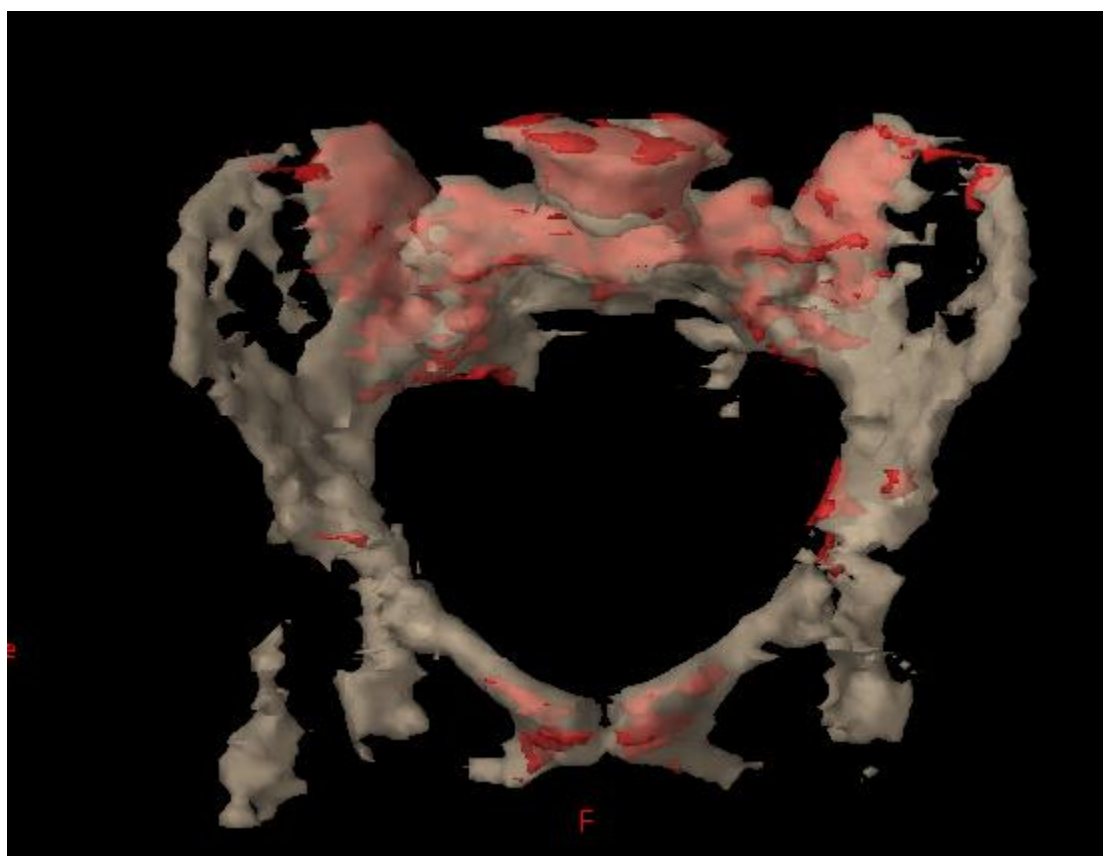

Supplement: Supplementary data 4 [file mmc4.pdf]
